# Does mosquito mass-rearing produce an inferior mosquito?

**DOI:** 10.1186/s12936-017-2012-8

**Published:** 2017-09-07

**Authors:** Dieudonné D. Soma, Hamidou Maïga, Wadaka Mamai, Nanwintoun S. Bimbile-Somda, Nelius Venter, Adel B. Ali, Hanano Yamada, Abdoulaye Diabaté, Florence Fournet, Georges A. Ouédraogo, Rosemary S. Lees, Roch K. Dabiré, Jeremie R. L. Gilles

**Affiliations:** 1 Institut de Recherche en Sciences de la Santé/Centre Muraz, BP 545 Bobo-Dioulasso, Burkina Faso; 2 Insect Pest Control Laboratory, Joint FAO/IAEA Division of Nuclear Techniques in Food and Agriculture, Vienna, Austria; 3 Université Nazi Boni, Bobo-Dioulasso, Burkina Faso; 4 Institut de Recherche Agricole pour le Développement (IRAD), Yaoundé, Cameroon; 5 Vector Control Reference Laboratory, Centre for Opportunistic, Tropical & Hospital Infections, National Institute for Communicable Diseases / Wits Research Institute for Malaria, School of Pathology, Faculty of Health Sciences, University of the Witwatersrand, Johannesburg, South Africa; 6 Institut de Recherche pour le Développement (IRD), MIVEGEC, BP 64501, 34394 Montpellier Cedex 5, France; 7 Liverpool Insect Testing Establishment (LITE), Vector Biology Department, Liverpool School of Tropical Medicine, Pembroke Place, Liverpool, L3 5QA UK

**Keywords:** *Anopheles arabiensis*, Sterile insect technique, Mass-rearing, Competitiveness

## Abstract

**Background:**

The success of the sterile insect technique depends, among other things, on continuous releases of sexually competitive sterile males within the target area. Several factors (including high rearing density and physical manipulation, such as larvae and pupae separation) can influence the quality of males produced in mass-rearing facilities. The different steps in mass production in the laboratory may modify the behaviour of mosquitoes, directly or through loss of natural characters as a result of adaptation to lab rearing, and lead to the competitiveness of sterile male being reduced. In the present study, the objective was to evaluate the effect of mass-rearing conditions on sterile male sexual competitiveness in semi-field cages compared to routine small scale laboratory rearing methods.

**Methods:**

*Anopheles arabiensis* immature stages were reared both on a large scale using a rack and tray system developed by the FAO/IAEA (MRS), and on a small scale using standard laboratory rearing trays (SRS). Mosquito life history traits such as pupation rate, emergence rate, adult size as well as the effect of irradiation on adult longevity were evaluated. Moreover, 5–6 day old mosquitoes were released into field cages and left for two nights to mate and the mating competitiveness between sterile mass-reared males and fertile males reared on a small scale when competing for small scale reared virgin females was investigated. Resulting fertility in a treatment ratio of 1:1:1 (100 irradiated males: 100 non-irradiated males: 100 virgin females) was compared to control cages with 0:100:100 (non-irradiated control) and 100:0:100 (irradiated control).

**Results:**

No significant differences in life history parameters were observed between rearing methods. The competitiveness index of mass reared males (0.58) was similar to males reared on a small scale (0.59). A residual fertility rate of 20% was observed in the irradiated control (100:0:100), measured as the percentage of eggs collected from the cages which developed to adulthood. No significant difference was observed (t = 0.2896, df = 4, *P* = 0.7865) between the rearing treatments (MRS and SRS) in the fertility rate, a measure of mating competitiveness.

**Conclusions:**

The results showed that the FAO/IAEA mass-rearing process did not affect mosquito life history parameters or the mating competitiveness of males.

## Background

Malaria remains a serious threat to world health, causing an estimated 212 million malaria cases and 429,000 deaths in 2015 [1]. With no vaccine available, current malaria control strategies are mainly based on the use of insecticides, chemoprevention and case management [1]. Vector control has been and continues to be an effective means of disease control [1, 2], though the current malaria control strategies are being undermined by the rapid spread of resistance to common insecticide classes in major malaria vectors [3–6] and of *Plasmodium* to available anti-malaria drugs [7]. Therefore, innovative and/or alternative strategies are needed for more effective vector control [8, 9].

The sterile insect technique (SIT), involving release of males sterilized using gamma or X-rays, could be used in the context of integrated vector control as it has already been by many control programmes of insect pests or disease vectors (as reviewed by Lees et al. [10]). The success of the SIT component depends, among other things, on continuous releases of sexually competitive sterile male within the target area [11]. It is desirable that the released males be as sexually competitive as wild males [10]. The different processes of mass production in the laboratory may modify the behaviour of mosquitoes, by directly impacting their quality or over time through loss of natural characteristics during adaptation to lab rearing [12]. This can contribute to reducing the competitiveness of the sterile males, in addition to the impact of irradiation that can affect competitiveness when too high doses are used or handling methods are not optimized [13]. The rearing history of the colony and a lack of genetic diversity induced by the laboratory colonization might also alter male sexual vigour under field conditions [14]. Therefore, studies in semi-field cages are necessary to better evaluate the competitiveness of sterile mass-reared males and to determine the minimum required release ratio of sterile male to wild male that could impact wild insect populations.

The semi-field cage system provides a very valuable measure of these parameters, as demonstrated in previous studies in *Anopheles coluzzii* [15] and *Anopheles arabiensis* [16, 17]. However, all mosquitoes used in these previous experiments were reared using routine laboratory equipment at a small scale and no studies have assessed the competitiveness of sterile males reared using the FAO/IAEA mass-rearing unit.

This study aimed to assess the effect of mass-rearing conditions on mosquito life history traits and sterile male sexual competitiveness in semi-field cages compared to routine small scale laboratory rearing methods. The efficacy of the sterilization process was evaluated in terms of residual fertility which was further assessed when full sterilization was not reached by observing whether the larvae that hatched can reach adulthood.

## Methods

### Mosquito strain and rearing conditions

The *Anopheles arabiensis* Dongola strain, originating from the northern state of Sudan was used in all experiments. Adults were reared at a temperature of 27 ± 1 °C, 60 ± 10% relative humidity (RH) and maintained under a light regime (light: dark) of 12:12 h including 1 h each of dusk and dawn. Adult mosquitoes were mass-reared using large cages (200 × 100 × 20 cm) [18] loaded with around 15,000 pupae where emerging adults has access to 5% sugar solution using a Whatman filter paper (58 × 58 cm). Females were offered defrosted bovine blood in the Hemotek membrane feeder (Discovery Workshops, UK) [19] for 2 h. Eggs were collected from large cages and quantified according to methods described by Maïga et al. [20], and reared to adulthood on either a mass-rearing scale (mass-rearing MRS) or a small rearing scale (SRS) for all experiments.

### Mass-rearing scale

Immature stages were reared in the larval mass-rearing unit, a tiltable steel rack holding 50 trays (100 × 60 × 3 cm) developed at the FAO/IAEA IPCL [21]. Each tray was filled with 4 L of deionized water the day before adding the eggs to allow the water to reach room temperature (28–30 °C). An aliquot of 4000 eggs was dispensed into a plastic ring floating on the surface of the water in each of the fifty rearing trays. The IAEA larval diet (1%) was used to feed larvae [22], and pupae were collected by tilting the rack and separating them from remaining larvae following the IAEA guidelines [23].

### Small rearing scale

Aliquots of 750 eggs obtained from mass-rearing cages were hatched and reared in small plastic laboratory trays (30 × 40 × 7 cm) filled with 1 L of deionized water [24]. A 1% solution of the IAEA diet was used to feed larvae: 10 mL per tray for the first 3 days, 20 mL on the 4th day and 30 mL on each remaining day as described by Mamai et al. [24]. Pupae were removed on a daily basis using a pipette, counted and placed into small bowls containing 50 mL of the same water treatment as they had been reared as larvae to homogenize rearing conditions. Pupae collected from each rearing method were divided into two groups, one for irradiation and one for adult size and longevity measurements.

### Irradiation of pupae

Pupae collected from both rearing conditions were separated by sex under a stereomicroscope by observing the shape of their genitalia [25]. Individuals pupating between 9:00 a.m. and 3:00 p.m. each day were collected for irradiation at 11:00 a.m. the following day, so that 20–26 h old male pupae were irradiated with gamma rays generated by a Cobalt-60 Gammacell^®^ (Nordion 220) source at the IPCL (Seibersdorf, Austria) at a dose of 75 Gy. To avoid possible variability related to radiation exposure, pupae originating from MRS and SRS were irradiated at the same time, 75 pupae per batch with most of the rearing water removed. The precise dose received by pupae in each treatment was measured with a dosimetry system using Gafchromic^®^ HD-810 film (International Specialty Products, NJ, USA) [23]. After irradiation, pupae from each replicate were separated into small cages (30 × 30 × 30 cm, Bugdorm 1H; Mega View, Taiwan), allowed to emerge overnight, and adults given access to 5% sucrose solution.

### Adult size

Wing lengths were measured as a proxy for adult size [26, 27]. Right wings were dissected, placed on a microscope slide and an image of the wing taken using a digital camera mounted on a stereo microscope. The wing length, defined as the distance from the axillary incision (alula) to the apical margin (excluding fringes), was measured from the digital images using analysis_FIVE software (Soft Imaging System, Germany). Wing lengths from 154 male mosquitoes were used to compare SRS and MRS treatments (about 35–40 wings from each of four replicates).

### Adult longevity

The longevity of newly emerged males was assessed (50 unirradiated and 50 irradiated) in small rearing cages (30 × 30 × 30 cm) with females kept under standard insectary conditions (27 ± 1 °C and 60 ± 10% RH) for the duration of the experiment. A 5% sucrose solution was supplied in a 150 mL plastic bottle with a filter paper. Dead mosquitoes were removed daily and counted until the last individual died. For each group, three replicates were performed.

### Male mating competitiveness

**Fig. 1:**
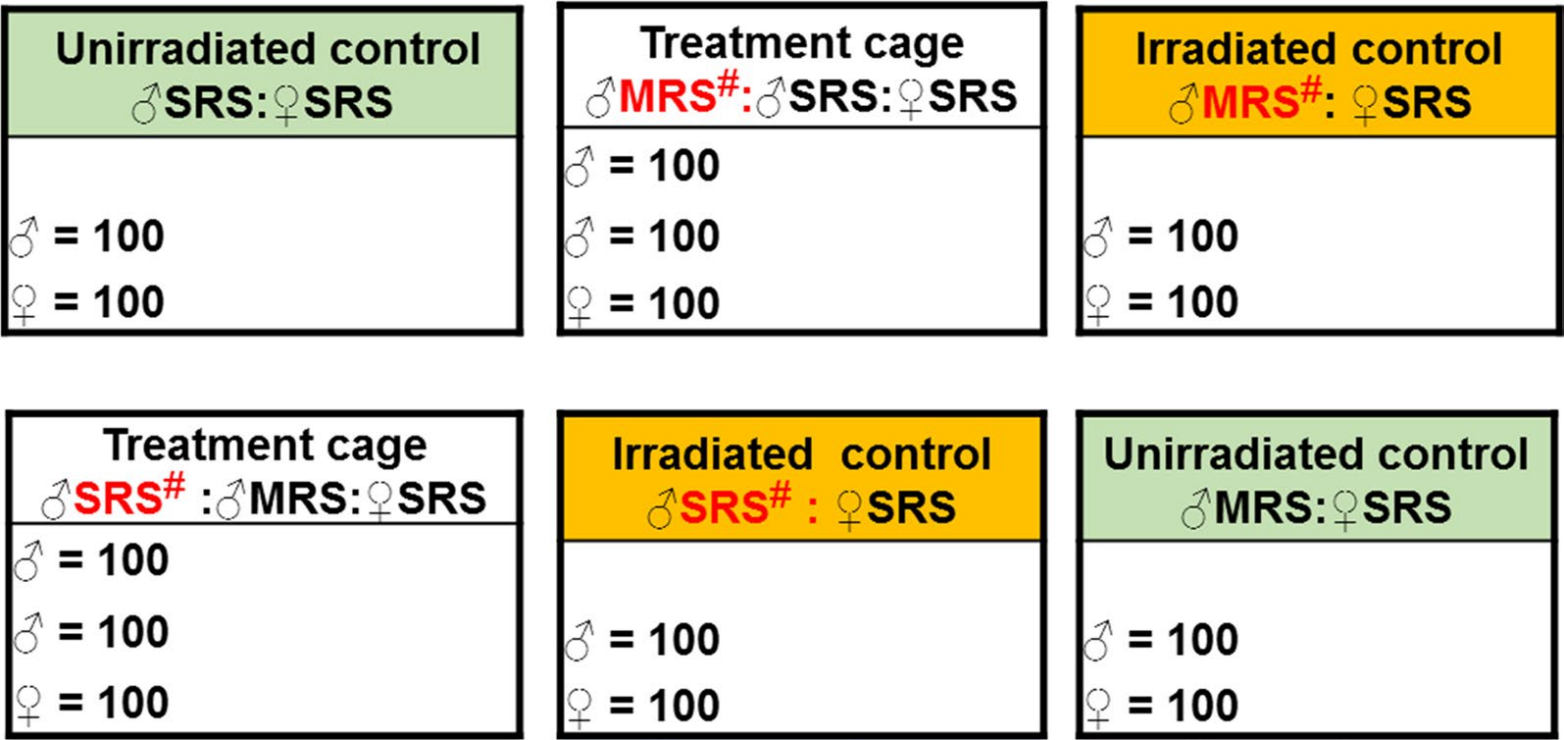
Experimental design used to assess *Anopheles arabiensis* male mating competitiveness. *MRS* mass-rearing scale, *SRS* small rearing scale, ^#^irradiated males; ♀ female; ♂ male

Experiments were conducted in semi-field cages (1.75 × 1.75 × 1.75 m, 5.36 m^3^, Live Monarch. Boca Raton. USA) in a climate controlled greenhouse (average temperature of 27 ± 1 °C, 50 ± 5% RH and natural light). A larval tray (100 × 60 × 3 cm) was introduced into each cage containing two 150 mL plastic bottles of 5% sucrose solution with a filter paper (Melitta 1 × 4 ORIGINAL FSC C095206). The trays served as resting sites and as attractants, facilitating the location of the sugar sources by the mosquitoes [15–17]. Five to 6 day old mosquitoes were released into field cages and allowed to mate for two nights in a treatment cage containing 1:1:1 (100 irradiated males: 100 non-irradiated males: 100 virgin females) or control cages containing either 0:100:100 (non-irradiated control) or 100:0:100 (irradiated control). Five replicates of each treatment were randomly positioned within the greenhouse (Fig. 1). All virgin females used in this competitiveness experiment provided from SRS and the males either from MRS or SRS.

On the 3rd day following release, all females were recollected from the field cages with a mouth aspirator and placed inside 30 × 30 × 30 cm cages. Defrosted bovine blood in the Hemotek system [19] was used to feed females for 30 min on each of three consecutive days. Blood fed females were allowed to lay eggs *en masse* in plastic cups (90 mm diameter) containing a filter paper (Cat No. 1001 090. 597; Whatman^®^, Maidstone, UK) on a wet sponge cloth soaked in water until fully saturated. Eggs were collected daily for 5 days and each batch was allowed to hatch over 2 days [15].

The number of pupae and adults resulting from eggs collected from treatment and control cages were used to calculate the fertility of each. Pupation rate and emergence rate were calculated by dividing the number of pupae and emerging adults by the number of collected eggs from each treatment. Induced sterility (IS) [28] was calculated following the method used by Yamada et al. [16]. Insemination rate was assessed after the oviposition period by dissecting the spermatheca of the recaptured females under a stereomicroscope. Females that died before oviposition were also dissected. The presence/absence of spermatozoa was confirmed using a compound light microscope at 400× magnification.

### Parameters measured and statistical analysis

The mean number of eggs laid per cage (females recaptured from semi-field cages) was counted using a stereomicroscope and a mean fecundity for each treatment was calculated by dividing the number of eggs laid daily by the number of females still alive (before egg collection) [15]. The mean insemination rate was also used to estimate the average number of females that laid eggs and the average number of eggs laid per female. Egg hatch rate (fertility) was assessed by dividing the number of first instar larvae (L1) counted by the number of eggs laid. After hatching and being counted, larvae were transferred into plastic trays (30 × 40 × 7 cm) and reared according to the protocol developed by Mamai et al. [24].

The competitiveness index (C) described by Fried [29] was calculated for each treatment using egg hatch rate from the unirradiated control (Ha), irradiated control (Hs) and competitiveness treatments (Ho) as follows: C = ((Ha–Ho)/(Ho–Hs)) × (N/S); where N is the number of unirradiated males and S the number of irradiated males.

Graphics were produced and all statistical analyses were performed using Microsoft Excel 2013 (Microsoft^®^, USA) and Graph Pad Prism v.5.0 software. Fecundity, egg hatch rate, insemination rate, pupation rate and emergence rate were analysed using analysis of variance (ANOVA) with Tukey’s honestly significant difference (HSD) post hoc tests. Wing length was tested for normality with the Kolmogorov–Smirnov test and mean length of male and female mosquitoes obtained from SRS and MRS compared using the Student’s *t* test. The Kaplan–Meier method was used to assess adult longevity. All data expressed as a proportion were arcsine-square-root transformed to stabilize variance and normalize distribution before analysis. The alpha level was *P* < 0.05.

## Results

### Adult size

**Fig. 2:**
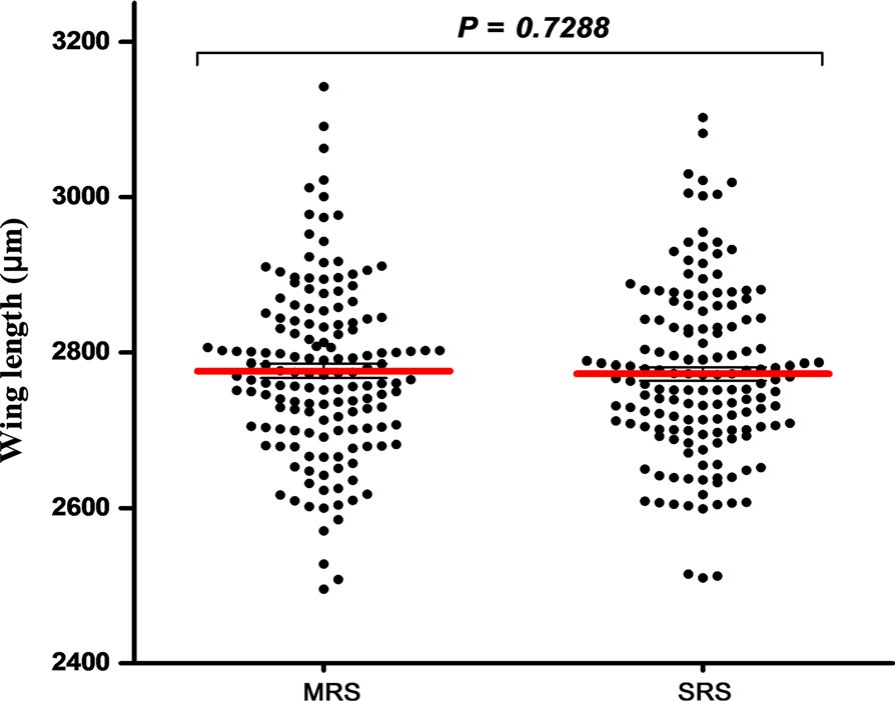
Mean wing length in *Anopheles arabiensis* reared at MRS and SRS

No significant difference was observed when wing lengths of males from the SRS treatment (n = 154) were compared to those from the MRS treatment (n = 156) group (t = 0.3473, df = 152, *P* = 0.7288) (Fig. 2).

### Adult longevity

**Fig. 3:**
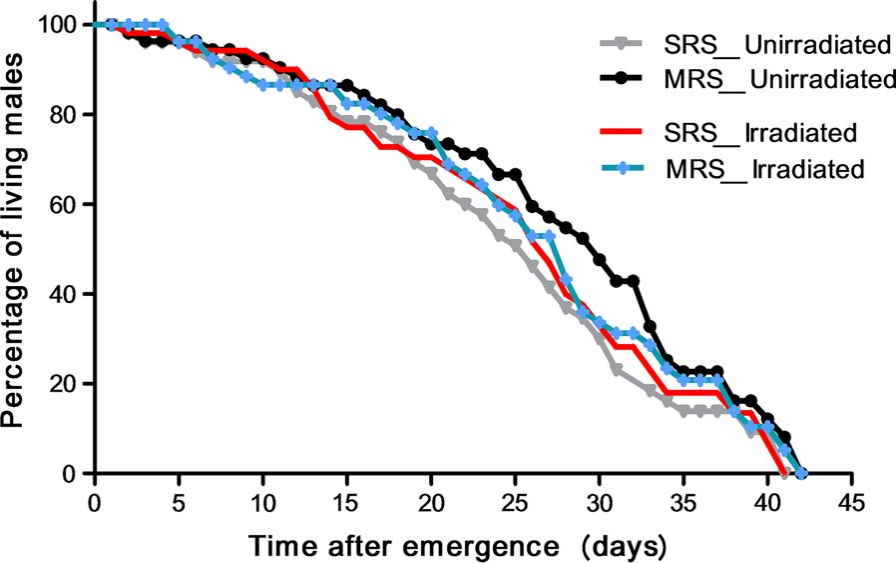
Longevity curves of *Anopheles arabiensis* males reared at MRS and SRS

A Log-Rank (Mantel-Cox) test comparison of longevity (Fig. 3) showed no significant difference in longevity between irradiated or unirradiated males either from SRS or MRS (χ^2^ = 2.473, df = 3, *P* = 0.4801). Time to 100% mortality was 41 days for SRS males and 42 days for those from MRS, a difference that was not statistically significant (Log-rank (Mantel-Cox) test, χ^2^ = 0.6782, df = 1, P = 0.4102).

### Recapture rate

The average percentage of females recaptured from semi-field cages after 2 days of mating ranged from 68.20 to 81.28% across all treatments (Table 1). No statistically significant difference in female recapture rate between treatment and control cages (ANOVA, F = 0.8776, df = 5, *P* = 0.5059) was observed.

**Table 1:** Mean proportion of recaptured females, female insemination rate and fecundity of *An. arabiensis* following mating competitiveness experiments in semi-field cages

Treatments	Ratio S♂:F♂:F♀	Mean of R ± SE (min–max)	IR		F (mean ± SE)
			n	% mean ± SE	
Unirradiated control-MRS	0:100:100	81.28 ± 3.81 (70–92)	212	64.30 ± 4.15	691.75 ± 177.46
Unirradiated control-SRS	0:100:100	76.20 ± 5.62 (54–84)	199	60.17 ± 4.36	234.50 ± 148.05
Irradiated control-MRS	100:0:100	68.20 ± 3.26 (59–76)	211	53.22 ± 5.85	370.75 ± 281.13
Irradiated control-SRS	100:0:100	73.83 ± 7.94 (49–94)	235	57.34 ± 5.30	598.50 ± 337.80
♂ MRS^#^:♂SRS:♀SRS	100:100:100	75.08 ± 6.23 (57–97)	280	53.84 ± 2.40	155.00 ± 89.83
♂SRS^#^:♂ MRS:♀SRS	100:100:100	72.43 ± 4.12 (57–89)	271	50.02 ± 1.47	206.00 ± 55.75

*R (%* *±* *SE)* average percentage of recaptured females, *(min–max)* minimum and maximum, *IR (%* *±* *SE)* proportion of dissected females per treatment, *n* number of dissected females, *F (%* *±* *SE)* average number of eggs laid per female, *SE* standard error, *S♂* irradiated male, *F♂* unirradiated male, *F♀* virgin female

### Insemination rate and fecundity

Insemination rate did not differ significantly between control and treatment cages (ANOVA, F = 0.1831, df = 5, *P* = 0.9663). The number of eggs collected from different treatments varied from 155.00 to 691.75 (Table 1); but no significant difference between treatment and control cages was observed (ANOVA, F = 1.137, df = 5, *P* = 0.3733).

The number of eggs per female, calculated based on insemination rate and total number of eggs, was 49.32 ± 28.23 and 12.69 ± 7.28 for unirradiated control MRS and SRS treatments, respectively (Tukey Multiple Comparison Test, P > 0.05). Females mated with irradiated males (in the sterile control) from the MRS treatment laid 16.68 ± 9.90 eggs and 29.22 ± 19.95 eggs when mated to irradiated males from the SRS (Tukey Multiple Comparison Test, P > 0.05). Females from treatment cages where males were in competition (irradiated males from MRS competing with unirradiated SRS males, and irradiated males from SRS competing with unirradiated MRS males) laid 6.24 ± 2.70 and 19.85 ± 4.94 eggs, respectively (A Tukey Multiple Comparison Test, P > 0.05).

### Competitiveness index, egg hatch rate and induced sterility

The competitiveness indices of irradiated males compared to controls calculated from treatment cages at 1:1:1 ratio (irradiated males: unirradiated males: virgin females) were 0.58 ± 0.10 and 0.59 ± 0.07 for irradiated MRS and SRS males, respectively (Table 2). The egg hatch rate in treatment cages was not significantly different (t = 0.5145, df = 4, *P* = 0.6340). Egg hatch rates were significantly different (ANOVA, F = 1.179, df = 5, *P* = 0.3484) between control cages (unirradiated and irradiated) and competition treatment cages. The induced sterility was similar in 1:1:1 ratio cages: 27.81 ± 3.79 and 27.94 ± 2.21% in MRS and SRS male treatments, respectively.

**Table 2:** Competitiveness index of irradiated *Anopheles arabiensis* males and induced sterility in large cage experiment

Treatments	Ratio S♂:F♂:F♀	Hatch rate (% mean ± SE)	C (mean ± SE)	IS (% ± SE)
Unirradiated control-MRS	0:100:100	84.22 ± 2.88^a^		
Unirradiated control-SRS	0:100:100	86.22 ± 2.70^a^		
Irradiated control-MRS^#^	100:0:100	19.14 ± 1.45^b^		
Irradiated control-SRS^#^	100:0:100	20.85 ± 0.77^b^		
♂ MRS^#^:♂SRS:♀SRS	100:100:100	61.07 ± 2.32^c^	0.58 ± 0.10	27.81 ± 3.79
♂SRS^#^:♂ MRS:♀SRS	100:100:100	61.79 ± 2.05^c^	0.59 ± 0.07	27.94 ± 2.21

Significantly differences between hatch rates are indicated by different letters (Tukey’s posthoc test, *P* < 0.05)

*C* competitiveness index, *IS* induced sterility, *SE* standard error, *S♂* irradiated male, *F♂* unirradiated male, *F♀* virgin female

^#^Irradiated male

**Fig. 4:**
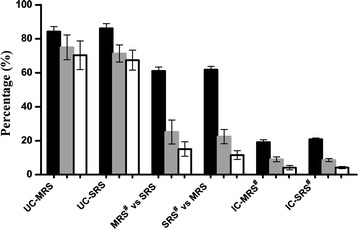
Proportion of *Anopheles arabiensis* larvae, pupae and adults emerging from eggs collected from different treatment cages. Proportion of larvae = black bars; pupae = grey bars; adults = white bars; *UC-MRS/UC-SRS* unirradiated control male from mass or small rearing scale, *IC-MRS/IC-SRS* irradiated control male from mass or small rearing scale, *MRS* ^*#*^ *vs SRS/SRS* ^*#*^ *vs MRS* irradiated male versus unirradiated male. Within each parameter were found not to be significantly different from each other (Tukey’s posthoc test, P < 0.05)

Overall, pupation rate from eggs collected from experimental cages was significantly higher in controls (unirradiated and irradiated) cages compared to the treatment cages (ANOVA, F = 3.778, df = 5, *P* = 0.0115). However, when the average pupation rate was compared between the rearing treatments (MRS and SRS), no significant difference was observed (t = 0.2896, df = 4, *P* = 0.7865). Similar observations were made in emergence rate in eggs collected from treatment cages (t = 0.5968, df = 4; *P* = 0.5828) (Fig. 4).

## Discussion

Rearing conditions have been shown to play a vital role in adult competitiveness of fruit flies and tsetse flies [30, 31], among other insect species. In this study, a comparative approach was employed to assess two rearing types in terms of their effect on a number. Life history traits of *An. arabiensis* and male mating competitiveness in semi-field cages.

In this experiment, neither body size nor longevity were adversely affected by the mass-rearing process compared to the small scale rearing routinely used. Although, the rearing was done for one generation, it would be important for further analysis to evaluate these parameters over multiple generations. The combination of rearing at high larval density and the processes of tilting and larvae/pupae separation of thousands of mosquitoes during mass-rearing did not appear to adversely affect the adults. This result is interesting because for SIT release programme to be successful, sterile males must be of sufficient quality to disperse into the environment, survive long enough to locate and attract wild females, in competition with wild counterparts, and copulate with as many as wild females as possible. The size of adult male mosquitoes is thought by many to be an important predictor of their mating success [15, 32–34]. Pupae of the same age and adult males of the same size were used here in order to minimize any effect of adult size and age on the mating competition between males from the SRS and MRS treatments. The observed variability in fecundity across all treatments and the relatively low number of eggs produced by the females could be attributed to female specific factors, such as the success of insemination or the volume of blood meal taken, which are known to affect insect fecundity [35]. The *en masse* egg collection method could partly explain the differences observed in the average number of eggs laid per female between treatments because it cannot take into account the exact number of females that laid, thus making estimates very approximate, as noted previously [36].

The face that neither adult body size nor longevity were significantly different between rearing treatments indicates that there was no major difference in the overall quality of males produced by these two rearing systems (MRS and SRS). Laboratory reared males may be less competitive than wild males, even without considering the damage caused by sterilization techniques, but there is a lot of controversy around how much of an impact colonization and mass-rearing has on subsequent mating success (see for example [37]). However, the results of these semi-field assays used to compare rearing treatments (SRS and MRS) reported in this study suggest that male competitiveness was not impacted by mass scale rearing.

A release ratio of 1:1 irradiated to fertile males induced about 27% of sterility, suggesting that in practice a higher ratio of irradiated to unirradiated males will need to be released to cause significant population reduction [38–40]. It is important to note that although the number of larvae hatching from cages where irradiated males were competing for mates was high, only half of larvae that hatched reached pupation and less than 20% survived to adult emergence. The apparently low competitiveness and high residual fertility of sterile males was therefore not as high as it first seemed, which is reassuring for the predicted success of releases of these males in an SIT programme. A competition experiment conducted in the same conditions as in this study by Yamada et al. [16] has shown that a 10:1 release ratio of *An. arabiensis* genetic sexing strain “ANO IPCL1” irradiated with a dose of 75 Gy and competing with fertile counterparts that would be necessary to induce about 80% sterility [17]. Munhenga et al. [17] have recorded a competitiveness index of 0.36 for an *An. arabiensis* strain which has been lab reared since 2010 while an *An. coluzzii* strain colonized in the lab for about 6 years has shown a competitiveness index of 0.53 under semi-field conditions [15].

In addition to rearing conditions and irradiation, inbreeding among laboratory reared mosquitoes negatively impacts a variety of male reproductive traits (for example, sperm vigor and size of mating plugs) which are crucial to their reproductive success [40]. It is therefore good practice in operational SIT programmes to refresh laboratory-kept colonies with field collected mosquitoes on a regular basis in order to reduce the effect of colonization and inbreeding on mating competitiveness [17].

## Conclusions

In this study, males reared under mass and small rearing conditions were compared, and mass production conditions (high population density, close space, tilting, larvae and pupae separation) were found not to affect mosquito life history traits, male competitiveness or induced sterility. The current technology and protocols developed at the FAO/IAEA IPCL for *An. arabiensis* mass-rearing are thus apparently adequate and ready to be implemented in SIT programmes. However, it is important in the context of SIT programmes to further assess the competitiveness of colonized and mass-reared males in semi-field conditions with females and males from wild-collected larvae or eggs to more accurately predict their performance after release. Other procedures involved in mass-rearing and production for release, including packing, chilling and handling before releases, not included in the scope of this study, also need to be investigated for their impact on male quality.

## Abbreviations

ANOVA: analysis of variance
FAO: Food and Agricultural Organization
IAEA: International Atomic Energy Agency
IPCL: Insect Pest Control Laboratory
LD: light: dark
MRS: mass-rearing scale
SIT: sterile insect technique
SRS: small rearing scale
RH: relative humidity
WHO: World Health Organization
